# Editorial: Advances and methods in mesenchymal stem cells

**DOI:** 10.3389/fcell.2024.1382019

**Published:** 2024-02-26

**Authors:** Pamela L. Wenzel

**Affiliations:** ^1^ Department of Integrative Biology and Pharmacology, McGovern Medical School, The University of Texas Health Science Center at Houston, Houston, TX, United States; ^2^ Center for Stem Cell and Regenerative Medicine, Brown Foundation Institute of Molecular Medicine, The University of Texas Health Science Center at Houston, Houston, TX, United States

**Keywords:** hematopoiesis, mesenchymal stem (stromal) cell, vitiligo, neural network models for morphology, fibro-adipogenic progenitor cell, neurotrauma and repair, antioxidant activity, mitochondrial transfer

Mesenchymal stem cells (MSCs) lie at the forefront of regenerative medicine and serve executive roles in maintaining homeostasis of the blood system. The articles in this Research Topic explore why MSCs have been so heavily exploited in clinical and basic science and address new tools and perspectives for understanding their differentiation and regenerative activities.

A major challenge in regenerative medicine is predicting how stem or progenitor cell differentiation will be impacted by the complex signals present at a site of injury. Li et al. tackle this problem in the context of chronic muscle injury, which is associated with muscle wasting, fibrotic scarring, and fat deposition attributed in part to pathogenic differentiation of muscle-resident interstitial progenitors. The authors develop a culture platform that integrates mixed signals more typical of an *in vivo* environment, as well as single signals, to interrogate the differentiation potential of various subsets of primary human muscle mesenchymal progenitors. Inclusion of the combination of myo-fibro-adipogenic signals enabled the identification of three progenitor subsets with varying intrinsic capacities for myogenic, fibrogenic, and adipogenic differentiation. Moreover, they leverage their platform to show that pharmacological control of gp130 signaling could prevent fibrosis and adipogenesis in fibro-adipogenic progenitor subsets while favoring enhanced myogenesis of myogenic subsets. This study reminds us that truly effective therapies will anticipate signals within the injured tissue to prevent pathological differentiation in conjunction with regeneration.

Even under culture conditions designed to direct differentiation toward a single lineage, fate commitment can be unpredictable or maturation incomplete. Classifying progeny often requires processing steps that kill the cells, rendering them unusable in functional assays. Various machine learning algorithms can predict lineage commitment with good accuracy using molecular signatures and microscopy of fixed cells. A few groups have recently reported that brightfield images can be used to train convolutional neural network models capable of determining cell type with reasonable accuracy. In Original Research, Mai et al. show that the morphology of live cells can be used to predict commitment of MSCs to adipocytes and osteoblasts with approximately 95% accuracy. Four neural network models were trained and tested head-to-head for their ability to classify differentiating MSCs captured by brightfield imaging. All models performed well, but ResNet 50 was found to excel in all metrics indicative of precision and accuracy. The authors suggest that the next steps for further improvement will include ensemble deep learning methods that can leverage the strengths of several models. If developed for integration with imaging systems, automated identification and annotation of live cell cultures could be a powerful tool for basic research and cell manufacturing.

The ability of MSCs to promote regeneration following injury has earned them a top seat among cellular therapies in clinical trials for neurotrauma. Using a culture-based model of central nervous system injury, de Laorden et al. explore the capacity of placental MSCs to promote regeneration, axonal growth, and functional restoration of adult axotomized retinal ganglion cells. The authors find that placental MSCs produce three growth factors known to stimulate axonal growth, reorganize and form synapses, and promote sensory neuron survival, namely, brain-derived neurotrophic factor (BDNF), nerve growth factor (NGF), and neurotrophin-3 (NT-3). Indeed, coculture of injured retinal ganglion cells with placental MSCs amplified the percentage of neurons with regenerated axons by 5–13 fold. Growth leading to axon elongation was directly tied to paracrine signaling by factors secreted by the MSCs, while neuron regeneration was dependent upon MSCs having direct contact with neurons. Interestingly, hypoxic culture elevated MSC secretion of NGF, suggesting that cellular therapy delivered to hypoxic regions could exhibit distinct profiles of growth factors that support different regenerative effects. Placental MSCs have attracted attention due to their ease of collection, abundance, plasticity, and immunomodulatory activity, and de Laorden et al. elegantly demonstrate the utility of this abundant tissue source for regeneration of cells damaged by neurological injury.

Within their native bone marrow niche, MSCs play executive roles in maintaining homeostasis of the hematopoietic system. In fact, accumulating evidence supports a central role for MSCs in diseases of the blood, including a process described as metabolic coupling by Singh et al. In their Mini Review, the authors discuss emerging research that demonstrates that metabolism of MSCs and hematopoietic cells are interconnected. These metabolic ties are exploited by numerous hematological malignancies but also impact outcomes following hematopoietic stem cell transplantation. MSCs and their progeny, adipocytes, modify metabolites in the niche and can transfer mitochondria. This metabolic crosstalk improves hematopoietic recovery after regenerative stress or transplantation but can also promote leukemia cell proliferation and contribute to chemoresistance. In addition to metabolic coupling, the authors provide timely updates on other mechanisms by which MSCs support hematopoiesis, including generation of factors important for preservation and expansion of hematopoietic stem cells, as well as detailed accounting of the functionally and phenotypically distinct subpopulations of bone marrow MSCs. Through this comprehensive Mini Review, the authors show that the metabolic coupling of bone marrow-resident MSCs and MSC therapeutics to hematopoietic cells presents a unique opportunity for hematological disease intervention.

Antioxidant activity is central to the ability of MSCs to promote hematopoietic recovery, as reported by Singh et al. In another Review focused on the MSC’s capacity for reducing oxidative stress, Yang et al. present a forward-looking perspective on MSCs as a treatment for vitiligo. Vitiligo is an autoimmune disorder that causes loss of the skin’s pigmentation. Melanocytes of affected individuals exhibit defects in peroxidase, mitochondria, and endoplasmic reticula which contribute to melanocyte destruction. The authors review current literature that point to oxidative stress as a key driver of vitiligo pathogenesis. Current therapies for vitiligo pose significant risk of complications and relapse, and the authors argue that MSC therapy should be explored as an alternative. MSCs could ameliorate the initiating oxidative stress thought to trigger vitiligo, as well as suppress autoimmune response and promote melanocyte regeneration. Yang et al. make a strong case for investment in future research to evaluate the safety and efficacy of MSC therapy in vitiligo.

